# Intensive Care Psychological Assessment Tool (IPAT): Turkish validity and reliability study

**DOI:** 10.3906/sag-1812-164

**Published:** 2019-08-08

**Authors:** Berker DUMAN, Zeynep KOTAN, Vahap Ozan KOTAN, Nevzat Mehmet MUTLU, Beyza DOĞANAY ERDOĞAN, Damla SAYAR AKASLAN, Safiye Zeynep TATLI, Hakan KUMBASAR

**Affiliations:** 1 Department of Psychiatry, Division of Consultation-Liaison Psychiatry, Faculty of Medicine, Ankara University, Ankara Turkey; 2 Department of Psychiatry, Dr. Abdurrahman Yurtaslan Ankara Oncology Training and Research Hospital,University of Health Sciences, Ankara Turkey; 3 Department of Psychiatry, Faculty of Medicine, Başkent University, Ankara Turkey; 4 Department of Anesthesiology and Reanimation, Ankara Numune Training and Research Hospital,University of Health Sciences, Ankara Turkey; 5 Department of Biostatistics, Faculty of Medicine, Ankara University, Ankara Turkey; 6 Department of Psychiatry, Faculty of Medicine, Ankara University, Ankara Turkey; 7 Department of Psychiatry, Division of Consultation-Liaison Psychiatry, Faculty of Medicine, Ankara University, Ankara Turkey

**Keywords:** Intensive care, anxiety, depression, delirium, validity, reliability

## Abstract

**Background/aim:**

It is of crucial importantance to be able to detect acute psychological distress in patients. The Intensive Care Psychological Assessment Tool (IPAT) was developed for this purpose in intensive care units. This study aims to evaluate the validity and reliability of the Turkish version of IPAT.

**Materials and methods:**

In total, 98 patients were included. To assess concurrent validity, the Intensive Care Experiences Scale (ICES) and the Hospital Anxiety Depression Scale were performed. Cronbach’s alpha coefficient was used to estimate internal consistency. Interitem and item-total score correlations were also performed. Sensitivity and specificity were derived for concurrent anxiety and depression.

**Results:**

The internal reliability was good. Cronbach’s a = 0.85. Items were well-correlated, with an average interitem correlation of 0.38. The concurrent validity of IPAT was good. Correlation between IPAT scores, anxiety, depression, ICES, and the diagnosis of delirium were as follows, respectively: r = 0.61, P < 0.01, r = 0.54, P < 0.01, r = −0.66, P < 0.01, r = 0.37, P < 0.01. With a cutoff score of ≥ 6, IPAT showed 85% sensitivity and 61% specificity to detect concurrent anxiety, and 74% sensitivity and 82% specificity to detect concurrent depression [AUC = 0.77 (95% CI, 0.68–0.87) and 0.84 (95% CI, 0.76–0.92), respectively].

**Conclusion:**

The Turkish version of IPAT was found to be a valid and reliable tool to assess acute psychological distress among patients in intensive care units.

## 1. Introduction

A high prevalence of psychiatric disorders have been reported in intensive care units (ICUs), independent from etiology [1]. Among the various psychiatric disorders presented, anxiety disorders, depression, and delirium were the most frequently encountered. In ICUs, prevalence rates of 13.7% for depression, 24% for anxiety disorders and 40%–80% for delirium were reported [2]. Morbidity and mortality rates have been found to be higher in intensive care patients with delirium as well as other psychiatric disorders [3,4]. With the help of advancing medical technologies, rates of ICU survival are increasing. As a result of traumatic experiences in ICUs, higher rates of anxiety, depression, and posttraumatic stress disorder were reported [5]. The psychological symptoms of patients in ICUs should thus be screened routinely, and when necessary interventions should be considered, as suggested by recent guidelines [6]. Such an approach promises to improve quality of life, reduce rates of morbidity and mortality of patients in ICUs, and may lower future risk of trauma-related psychiatric disorders [6]. However, evaluating patients’ psychiatric symptoms in an ICU is difficult, especially for patients currently on mechanical ventilator support [7,8]. For these purposes, several screening scales have been developed, yet until recently there has been no quick, easy-to-use tool available. In light of this, Wade et al. (2014) developed the ‘Intensive Care Psychological Assessment Tool (IPAT)’ as a simple, quick screening tool to detect acute psychological distress and the risk of future psychiatric morbidity in ICU patients. The IPAT was found to have good validity and reliability, and was acceptable, quick and easy for both ICU patients and medical staff to use. Negative experiences such as communication difficulties, sleep disorders, hallucinations, delusions, depressed mood, anxiety, and feelings of panic were evaluated by items on the IPAT [6]. 

In our study, we aimed to examine the validity and reliability of an adaptation of IPAT into Turkish as a means to detect acute psychological distress in intensive care patients in Turkey.

## 2. Materials and methods

### 2.1. Study design, setting, and recruitment sites

Our study was carried out in the surgical, medical, pulmonary, coronary, and reanimation ICUs of 2 training and research hospitals. All participants were intensive care patients who fulfilled the following inclusion criteria: they had been in an ICU for at least the last 48 h, were Turkish-speaking, over 18 years of age, literate, able to communicate, and were awake at the time of application of the questionnaire. Participants with a Glasgow Coma Scale (GCS) score below 15, who were delirious at the time of assessment, or who had any neurological or sensorial-motor dysfunction that might hinder the evaluation were excluded from the study. All patient psychiatric evaluations were carried out by experienced clinicians, and patients diagnosed with dementia, mental retardation, autistic spectrum disorder, bipolar disorder, schizophrenia, and alcohol or substance misuse were also excluded from the study. 

The study received approval from the local ethics committee (reference no: E16-730). After a description of the study to each participant, written and verbal informed consent was obtained. 

### 2.2. Measurements

#### 2.2.1. Sociodemographic and clinical data collection

In addition to sociodemographic variables, information about smoking history, alcohol and substance history, length of stay in the ICU, length of stay in the hospital, diagnosis, acute physiology, and chronic health evaluation II (APACHE II) scores; the type of ICU (surgical, medical, pulmonary, coronary, reanimation); the need for mechanical ventilator support; the number of ICU admissions; psychiatric history; current psychiatric therapy; psychiatric consultation; and delirium histories were obtained from all participants. Delirium has been diagnosed by using DSM-5 criteria [9].

#### 2.2.2. Intensive Care Psychological Assessment Tool (IPAT)

The Intensive Care Psychological Assessment Tool (IPAT) was developed by Wade et al. (2014). The IPAT is a 10-item, 3-category Likert scale (‘no’, ‘yes, a bit’, and ‘yes, a lot’), scored as 0, 1, and 2. When applying the scale, evaluators helped patients in the answering process, such as showing or reading the scale questions and choices. The questionnaire was developed for 2 purposes: to detect acute psychological distress in ICU patients, and to predict patients at risk of future psychiatric morbidity. Because none of the existing questionnaires functioned well as quick screening tools, the research team who developed the IPAT aimed to create something easily administered by doctors and bedside nurses alike, and simple enough to be understood by critically ill patients. Higher scores indicate negative intensive care experiences. The test-retest reliability was good (r = 0.8), as was concurrent validity with measures of anxiety and depression (respectively r = 0.7, P < 0.001; r = 0.6, P < 0.001). With a cut-point of ≥7, the IPAT showed 82% sensitivity and 65% specificity to detect concurrent anxiety, and 80% sensitivity and 66% specificity to detect concurrent depression. Also, predictive validity for psychological morbidity was good (r = 0.4, P < 0.01; r = 0.64, P < 0.01). The IPAT showed 69% specificity and 57% sensitivity to predict future psychological morbidity (AUC = 0.7). The IPAT was thus found to have good reliability and validity [6].

The translation of the scale into Turkish was carried out by 3 psychiatrists, blind to each other. After reaching a consensus, another psychiatrist translated the Turkish version back to English. The back-translation to English was then reviewed. The final Turkish version is provided in Table 1. A sample size was calculated, with plans to recruit 95–100 patients. 

**Table 1:** Turkish version of intensive care psychological assessment tool (IPAT). Size yoğun bakımda kaldığınız süre boyunca nasıl hissettiğinize ilişkin bazı sorular sormak istiyorum. Burada yaşadığınız duygular iyileşme süreciniz için önemli olabilir. Size en yakın gelen yanıtı işaretleyin ya da yapabileceğiniz herhangi bir yolla (örneğin konuşarak veya işaret ederek) cevap verin.

	Yoğun bakımda kaldığınız süre içinde:	A	B	C
1	İletişim kurmakta zorlandınız mı?	Hayır	Biraz	Çok fazla
2	Uyumakta zorluk çektiniz mi?	Hayır	Biraz	Çok fazla
3	Gergin hissediyor musunuz?	Hayır	Biraz	Çok fazla
4	Üzgün hissediyor musunuz?	Hayır	Biraz	Çok fazla
5	Paniğe kapılmış hissediyor musunuz?	Hayır	Biraz	Çok fazla
6	Karamsar hissediyor musunuz?	Hayır	Biraz	Çok fazla
7	Aklınızın karıştığı oldu mu (nerede olduğunuzu bilememe gibi)?	Hayır	Biraz	Çok fazla
8	Halusinasyonlarınız oldu mu (gerçekten orada olduğundan şüphe duyduğunuz görüntüler gördünüz mü veya sesler duydunuz mu)?	Hayır	Biraz	Çok fazla
9	İnsanların size kasten zarar verdiğini ya da kötülük yaptığını düşündünüz mü?	Hayır	Biraz	Çok fazla
10	Yoğun bakımla ilgili rahatsız edici anılar aklınıza geliyor mu?	Hayır	Biraz	Çok fazla

#### 2.2.3. Intensive Care Experience Scale (ICES)

The Intensive Care Experience Scale (ICES) was developed by Rattray et al. (2004) to evaluate patients’ ICU experiences [10]. A validity and reliability study of the Turkish version of the questionnaire was performed [8]. The Turkish version consists of a 5-category Likert scale with 19 items. The total score of the scale ranges from 19 to 95, with higher scores indicating positive ICU experiences [8, 10].

#### 2.2.4. Hospital Anxiety and Depression Scale (HADS)

The Hospital Anxiety and Depression Scale (HADS) was developed to detect depression and anxiety in general hospital settings [11]. A Turkish validity and reliability study of the scale was performed [12]. In this study, a cutoff score of 7 was found for a depression subscale (HADS-D), and a cutoff score of 10 was found for an anxiety subscale (HADS-A) [12].

### 2.3. Statistical analyses

#### 2.3.1. Assessment of psychometric properties 

A total of 98 patients were recruited to the Turkish validity and reliability study of the IPAT. Cronbach’s alpha coefficient was used to estimate internal consistency. Confirmatory factor analysis (CFA) was used to examine the validity of the IPAT scale. In order to evaluate whether the data would fit the proposed unidimensional model, a bifactor CFA for categorical data was applied with a weighted least (WLSM) c2 estimation with robust standard errors and mean and variance adjusted statistics. In order to assess the degree of fit between the model and the sample, the following goodness of fit indices were used: Comparative Fit Index (CFI >0.90: acceptable, >0.95: excellent); Tucker–Lewis Index (TLI >0.90: acceptable, >0.95: excellent)’ and root-mean-square error of approximation (RMSEA <0.08: acceptable, <0.05: excellent) [13]. Analysis was conducted using R 3.3.3, “lavaan” package was used to perform CFA [14,15].

Concurrent criterion validity was assessed using the Spearman’s correlation coefficient. We chose HADS anxiety, HADS depression, ICES total scores, and diagnosis of delirium as criterion measures. Sensitivity and specificity were derived for concurrent anxiety and concurrent depression in ICU, using coordinates on the receiver operating characteristic (ROC) curve and the best cut point on the IPAT scale identified by Youden’s J index. Areas under the curve (AUC) for ROC were given with a 95% confidence interval (CI). Concurrent anxiety and depression were determined by cutoff points of 10 and 7 from HADS anxiety and HADS depression scores [12].

## 3. Results

### 3.1. Patient characteristics

Descriptive variables for the 98 patients in the study are provided in Table 2. The mean age of participants was 59.9 years (SD 15.7, range 18–91). The majority of the participants (74.5%, n = 73) were married, and 25.5% were unmarried. The sex breakdown was 62.2% (n = 61) male and 37.8% female. When evaluated in terms of education levels, 13.3% (n = 13) of the patients were literate with less than 5 years of education, 32.7% (n = 32) were primary school graduates, 13.3% (n = 13) were secondary school graduates, 26.8% (n = 26) high school graduates, and 13.3% (n=13) university graduates. The frequencies of ICU type were as follows: surgical ICU 26.5% (n = 26), reanimation ICU 25.5% (n = 25), coronary ICU 23.5% (n = 23), pulmonary ICU 18.4% (n = 18), internal medicine ICU 6.1% (n = 6). APACHE-II scores were calculated in 69.4% (n = 68) of patients. The mean APACHE-II score was 11.22 (SD 6.8, range 2–37). Mechanical ventilation support was received in 19.4% (n = 19) of the patients. 23.5% (n = 23) of the patients reported that they were still smoking, 24.5% (n = 24) had used alcohol in the past, and 4.1% (n = 4) reported a history of substance abuse. 

**Table 2:** Sociodemographic and clinical data.

	n(%)mean ± SD (min–max)
Age	59.90 ± 15.69 (18–91)
Sex, F/M	37 (37.8%) / 61 (62.2%)
Type of ICUSurgicalReanimationCoronaryPulmonaryInternal medicine	26 (26.5%)25 (25.5%)23 (23.5%)18 (18.4%)6 (6.1%)
APACHE-II score (n = 68)	11.22 ± 6.75 (2–37)
Positive history of psychiatric disorder	22 (22.4%)
Current psychotrophic medicine use	16 (16.3%)
ICU- psychiatry consultation	16 (16.3%)
ICU-delirium diagnosis	20 (20.4%)
ICU-mechanical ventilator support	19 (19.4%)
Length of ICU stay (days)	7.37 ± 10.42 (median: 4.2–90)
IPAT total score	6.26 ± 4.48 (0–18)
ICES total score	62.93 ± 8.32 (41–80)
HADS-Anxiety score	8.48 ± 4.79 (0–20)
HADS-Depression score	9.35 ± 4.89 (0–19)

IPAT: Intensive Care Psychological Assessment Tool, ICES: Intensive Care Experiences Scale, HADS-Anxiety: Hospital Anxiety Depression Scale Anxiety Subscale Score, HADS-Depression: Hospital Anxiety Depression Scale Depression Subscale Score

The number of ICU admissions was collected. 63.3% (n = 62) of the sample reported that this was their first ICU stay, and 22.4% (n = 22) reported a second stay. 22.4% (n = 22) of the patients had a history of psychiatric diagnosis and treatment. 10.2% (n = 10) of the patients reported ongoing psychiatric treatment. 16.3% (n = 16) of the sample used at least 1 psychotropic medication at the time of ICU evaluation. The percentage of patients who met the diagnosis of delirium at least once during their current ICU stay was 20.4% (n = 20). The mean total length of stay in the ICU was 7.37 (SD 10.4, median 4, range 2–90) days. 

The IPAT total score was 6.26 (SD 4.5, range 0–18), the ICES 62.93 (SD 8.3, range 41–80), the HADS-A 8.48 (SD 4.8, range 0–20), and the HADS-D total score was 9.35 (SD 4.9, range 0–19) (Table 2).

### 3.2. Psychometric properties of the IPAT

#### 3.2.1. Reliability

Internal reliability was good (Cronbach’s a = 0.85). Items were well-correlated, with an average interitem correlation of 0.38; individual interitem correlations ranged from 0.12 to 0.70. The range of corrected item-scale correlations was 0.41 to 0.71. These results suggest reasonably good levels of internal consistency for the IPAT scale. Results are provided in Tables 3 and 4.

**Table 3:** Inter-item correlations of IPAT.

	IPAT_1	IPAT_2	IPAT_3	IPAT_4	IPAT_5	IPAT_6	IPAT_7	IPAT_8	IPAT_9	IPAT_ 10
IPAT_1	1.000	0.262	0.282	0.330	0.183	0.397	0.375	0.538	0.385	0.590
IPAT_2	0.262	1.000	0.361	0.356	0.123	0.286	0.290	0.207	0.368	0.275
IPAT_3	0.282	0.361	1.000	0.499	0.441	0.450	0.243	0.279	0.176	0.309
IPAT_4	0.330	0.356	0.499	1.000	0.622	0.700	0.347	0.280	0.229	0.306
IPAT_5	0.183	0.123	0.441	0.622	1.000	0.614	0.371	0.253	0.234	0.213
IPAT_6	0.397	0.286	0.450	0.700	0.614	1.000	0.523	0.394	0.237	0.431
IPAT_7	0.375	0.290	0.243	0.347	0.371	0.523	1.000	0.683	0.488	0.459
IPAT_8	0.538	0.207	0.279	0.280	0.253	0.394	0.683	1.000	0.565	0.621
IPAT_9	0.385	0.368	0.176	0.229	0.234	0.237	0.488	0.565	1.000	0.473
IPAT_10	0.590	0.275	0.309	0.306	0.213	0.431	0.459	0.621	0.473	1.000

IPAT: Intensive Care Unit Psychological Assessment Tool *By Spearmans’ correlations analysis

**Table 4:** Item-total score correlations of IPAT.

	Corrected item-total score correlations*
IPAT_1	0.530
IPAT_2	0.405
IPAT_3	0.525
IPAT_4	0.650
IPAT_5	0.525
IPAT_6	0.707
IPAT_7	0.608
IPAT_8	0.604
IPAT_9	0.498
IPAT_10	0.586

IPAT: Intensive Care Unit Psychological Assessment Tool * By Spearmans’ correlations analysis

#### 3.2.2. Validity

Ten items for the IPAT were subjected to bifactor CFA to confirm the unidimensional structures. According to factor loadings and goodness-of-fit statistics, these unidimensional structures were confirmed for the scale. The data showed a reasonable fit to the bifactor CFA model, in which CFI = 0.952, TLI = 0.933, and RMSEA = 0.127. None of the items had factor loading below 0.40, so that all kept in the model. Items and factor loadings are given in Table 5. The concurrent validity of IPAT was good with respect to correlations to selected criteria. Correlations between IPAT total scores, concurrent anxiety and depression measures, ICES scores, and diagnosis of delirium were r = 0.61, P < 0.01; r = 0.54, P < 0.01; r = −0.66, P < 0.01; r = 0.37, and P < 0.01, respectively. Correlations between IPAT anxiety scores, HADS-A scores, HADS-D scores, ICES scores, and diagnosis of delirium were r = 0.57, P < 0.01; r = 0.51, P < 0.01; r = −0.51, P < 0.01; r = 0.18, and P = 0.08, respectively. Correlations between IPAT depression scores, HADS-A scores, HADS-D scores, ICES scores and diagnosis of delirium were r = 0.60, P < 0.01; r = 0.51, P < 0.01; r = −0.56, P < 0.01; r = 0.29, and P < 0.01, respectively. Correlations between IPAT delirium scores, HADS-A scores, HADS-D scores, ICES scores, and diagnosis of delirium were r =0.39, P < 0.01; r = 0.41, P < 0.01; r = −0.58, P < 0.01, r = 0.45, and P < 0.01, respectively (Table 6).

**Table 5:** Factor loadings of items.

Domain	Items	Factor loadings
Anxiety	Gergin hissediyor musunuz?	0.693
	Paniğe kapılmış hissediyor musunuz?	0.807
Depression	Üzgün hissediyor musunuz?	0.880
	Karamsar hissediyor musunuz?	0.927
Delirium	Aklınızın karıştığı oldu mu (nerede olduğunuzu bilememe gibi)?	0.857
	Halusinasyonlarınız oldu mu (gerçekten orada olduğundan şüphe duyduğunuz görüntüler gördünüz mü veya sesler duydunuz mu)?	0.980
	İnsanların size kasten zarar verdiğini ya da kötülük yaptığını düşündünüz mü?	0.809
IPAT_Total	İletişim kurmakta zorlandınız mı?	0.725
	Uyumakta zorluk çektiniz mi?	0.506
	Yoğun bakımla ilgili rahatsız edici anılar aklınıza geliyor mu?	0.845
	Anxiety	0.921
	Depression	0.912
	Delirium	0.857

**Table 6:** Concurrent and criterion validity: correlation between IPAT scores, HADS-anxiety scores, HADS-depression scores, ICES scores and delirium diagnosis.

	HADS_anxiety	HADS_depression	ICES	Delirium diagnosis
IPAT_total score	0.61*	0.54*	−0.66*	0.37*
IPAT_anxiety score	0.57*	0.51*	−0.51*	0.18
IPAT_depression score	0.60*	0.51*	−0.56*	0.29*
IPAT_delirium score	0.39*	0.41*	−0.58*	0.45*

*P < 0.001; IPAT: Intensive Care Psychological Assessment Tool, ICES: Intensive Care Experiences Scale, HADS-Anxiety: Hospital Anxiety Depression Scale Anxiety Subscale Score, HADS-Depression: Hospital Anxiety Depression Scale Depression Subscale Score

#### 3.2.3. Sensitivity and specificity

With a cutoff point of ≥ 6, the IPAT demonstrated 85% sensitivity and 61% specificity to detect concurrent anxiety, and 74% sensitivity and 82% specificity to detect concurrent depression [AUC = 0.77 (95% CI 0.680.87) and 0.84 (95% CI 0.76–0.92), respectively].

## 4. Discussion

Psychiatric symptoms are common in patients treated in ICUs, and psychiatric symptoms should be regularly screened and, when necessary, intervened in. Until recently, the screening tools developed for this purpose have been relatively difficult to use by nontrained ICU health professionals, and often it has not been possible for fatigued patients to respond in ICU conditions. For these reasons, the IPAT was developed to be used easily and quickly by ICU staff in order to detect acute psychological distress in ICU patients and to predict patients at risk of future psychiatric morbidity [6]. The IPAT is easily responded to by intensive care patients in a few minutes. According to the results of our study, the Turkish version of the IPAT has been found to have good internal reliability, good concurrent validity and reasonable factor loadings. With a cutoff point of ≥ 6, the Turkish version of the IPAT was found to have reasonable sensitivity and specificity to detect concurrent anxiety and depression. The Turkish version of the IPAT may offer a tool for ICU medical staff to assess acute psychological distress among ICU patients. 

There are a number of limitations to our study. First, experienced psychiatrists conducted the Turkish validity and reliability studies; ICU medical staff did not take part in the data collection process. This requires consideration because the primary aim of this work is for ICU medical staff to use the tool in order to screen acute psychological distress in intensive care patients. However, the results strike us as generalizable because patients responded to questions directly, without the subjective evaluation of the rater. 

Secondly, one of the reliability assessment methods recommended in psychometric scale studies is the use of test retest reliability [16]. This method could not be performed in our study, as the constantly changing conditions of patients in ICUs made it difficult to evaluate. However, our study design and our findings provide sufficient evidence for reliability evaluation.

Another limitation of our study was that the predictive validity of psychological symptoms was not evaluated. Our study is crosssectional, so it is not possible to perform this evaluation. Future studies with prospective cohort design are needed.

The Turkish version of the IPAT is a valid and reliable tool to detect acute psychological distress in intensive care patients. The IPAT may be used in clinical applications and for research purposes.
